# Classroom Predictors of National Belonging: The Role of Interethnic Contact and Teachers’ and Classmates’ Diversity Norms

**DOI:** 10.1007/s10964-021-01430-2

**Published:** 2021-04-07

**Authors:** Lian van Vemde, Lisette Hornstra, Jochem Thijs

**Affiliations:** 1 Department of Interdisciplinary Social Science, Utrecht University, Padualaan 14, 3584CH Utrecht, The Netherlands; 2 Department of Education, Utrecht University, Heidelberglaan 1, 3584CS Utrecht, The Netherlands

**Keywords:** National belonging, Minority students, Multicultural teacher norms, Teacher closeness, In-group norms, Out-group norms

## Abstract

Schools can be important for the development of national belonging in students with immigrant backgrounds. Following Contact Theory and prior research on diversity norms, this cross-sectional survey study examined if intergroup contact and perceived diversity norms of teachers and classmates predicted national belonging in ethnic minority (i.e., Turkish [*n* = 95], Moroccan [*n* = 73], and Surinamese [*n* = 15]) versus majority students (*n* = 213) living in the Netherlands (*M*_age_ = 10.53 years; 50.3% female). Minority students reported less national belonging than their ethnic Dutch classmates. Multilevel analyses indicated that their national belonging was affected by the presence of ethnic Dutch classmates and the relationship with their teacher. These results indicate that minority students’ national belonging could be promoted by reducing school segregation and stimulating positive teacher-student relationships.

## Introduction

Children with an ethnic minority or immigrant background can experience a weak sense of belonging to (e.g., Fleischmann & Phalet, 2018), or even feel alienated from (Leeman & Saharso, 2013), the countries they live in. This may be a logical or even adaptive response to experiences of discrimination and marginalization (Kende et al., 2020) but it may also hamper their psychological well-being (Wu et al., 2018) and social adjustment (Berry et al., 2006). National belonging does not automatically imply assimilation to majority culture as immigrant children can feel connected to the “host” country yet still have a strong ethnic identity (e.g., Berry & Hou, 2016). Schools can be important places for the development and stimulation of national belonging, especially for ethnic minority children whose cultural background differs from that of the national majority group members (Spiegler et al., 2018). Still, very few studies (Agirdag et al., 2011) have examined how the school environment can contribute to (ethnic minority) children’s sense of national belonging. Using cross-sectional survey data, the present study tries to make a unique contribution to this emerging literature by examining whether Dutch primary school students (age 9–13 years) of immigrant descent felt a lower sense of belonging to the Netherlands compared to their ethnic Dutch^1^ classmates, and whether their national belonging could be predicted by the opportunity for contact with ethnic Dutch classmates, the quality of contact with their teachers, and the perceived diversity norms of their classmates and teachers.

### National Belonging

National belonging refers to the emotional involvement of people with the state they live in, and individuals who experience this belonging feel connected to, and at home in their country as well as close to their co-nationals (Ashmore et al., 2004). National belonging is the affective aspect of national identification (Ashmore et al., 2004) and considered to be a crucial component of citizenship (Kallio et al., 2020). If different groups feel connected to the country they live in, their members are more likely to be(come) “good citizens” and to contribute to the common good (Dovidio et al., 2007). Moreover, national belonging promotes national solidarity, social cohesion, and stability of diverse societies, as well as effective democracy (e.g., Putnam, 2007). National belonging is thus different from assimilation, as the latter means that ethnic minority people give up their heritage culture and fully adjust to the majority culture (Berry et al., 2006). Actually, research has shown that children with immigrant backgrounds develop both an ethnic and a national identity (Umaña-Taylor et al., 2014). Also, research suggests that for ethnic minority children to be able to engage in other cultures, such as the national culture, they first have to develop a secure and stable sense of belonging to their ethnic group (Phinney et al., 2007). This means that someone can experience a sense of belonging to both groups and that these types of belonging do not have to come at each other’s expense. In fact, it is the combination of both, often referred to as integration, that has proven to be beneficial for the socio-cultural and psychological adaptation of ethnic minority youth (Berry et al., 2006).

Studies in several Western countries have shown that (some groups of) ethnic minority people experience a lower sense of national belonging compared to the ethnic majority group members (e.g., Elkins & Sides, 2007). This seems to hold especially for ethnic minority groups in so-called non-settler countries (i.e., “older” nations with an entrenched indigenous majority population, such as the Netherlands and Germany), compared to settler countries (i.e., nations founded on a long immigration history like the United States and Australia; Simonsen, 2016). In these non-settler countries the national label (e.g., “Dutch”) is often used to refer to the ethnic majority population only. Consequently, the use of hyphenated or dual identities referring to both the ethnic and national identity (e.g., “Moroccan-Dutch”) is less accepted and sometimes even considered problematic by ethnic majority group members (see Verkuyten & Martinovic, 2012). The ethnic, heritage-based representation of who is a national in those non-settler countries can make it more difficult for immigrant and ethnic minority groups to feel included and experience a sense of belonging there (Verkuyten & Martinovic, 2012).

Although previous research on national belonging has primarily focused on ethnic minority adults (e.g., De Vroome et al., 2014), it is important to study it in ethnic minority youth as well. Research in Western Europe has shown that both ethnic minority and ethnic majority children start to categorize themselves as national group members by the age of 5 or 6 (Barrett, 2002), that national identification can be reliably measured in 8-year-olds already (Oppenheimer, 2011), and that children are able to characterize their own and other national groups (Reizábal et al., 2004). Moreover, after middle childhood, the way children evaluate groups becomes increasingly context-dependent (Rutland et al., 2010), and this implies that preadolescence is an appropriate period for stimulating a positive national identity.

There are some indications that ethnic minority youth experience a lower sense of national belonging than ethnic majority youth (e.g., Phinney et al., 2006). This is unfortunate as national belonging is not only important for their lives as future adult citizens but could also affect them more directly. Research in the United States and Western Europe has shown, for example, that national belonging promotes ethnic minority youth’s educational achievement (Altschul et al., 2006), school adjustment (Motti-Stefanidi et al., 2008), and psychological well-being (Wu et al., 2018). However, it is reasonable to assume that national belonging can be fostered in the school context. Schools are public spaces where students from different backgrounds are taught to learn and work together, and prepared to participate as future citizens in societies that are increasingly diverse (Parker et al., 2008). Moreover, apart from being potential acculturation contexts where students learn about other ethnic groups and their respective cultures, schools are also institutions of national and civic enculturation where students are taught about the history, political system, and traditions of the country they all live in (see Barrett, 2007).

The present study focused on how the school context could contribute to the national belonging of students of Turkish, Moroccan, and Surinamese descent in the Netherlands. Almost all of these children are Dutch nationals but their immigrant backgrounds set them apart from the “original” inhabits of the Netherlands (i.e., ethnic Dutch). Turks, Moroccans, and Surinamese constitute the largest non-Western minority groups in the Netherlands. The presence of the first two groups is mainly due to labor migration during the 1960s and 1970s and which resulted in further family reunion in the 1980s and 1990s (De Vroome et al., 2014). Surinamese people mainly immigrated after Surinam, a former colony of the Netherlands, became independent in 1975 (Van Meeteren et al., 2013). Although there are many differences between Turks, Moroccans, and Surinamese in the Netherlands, all of these groups have a minority status in the country. That is to say, they face discrimination (for example on the labor market; Andriessen et al., 2012) and have, on average, lower educational attainment compared to ethnic Dutch people (De Vroome et al., 2014).

### Contact and Contact Opportunity

According to *Intergroup Contact Theory* (Allport, 1954), positive contact with members of a particular out-group (i.e., another group than one’s own) can promote positive feelings toward that group as a whole (for a meta-analysis, see Pettigrew & Tropp, 2006). One of the explanations for this generalization effect is that positive intergroup contact makes the out-group (“them”) less different from the in-group (“us”) and thereby stimulates the development of a common identity, that is to say a sense of belonging to a shared group (see the *Common In-Group Identity Model*; Dovidio et al., 2007). In case of contact between immigrants and ethnic majority populations, this common identity can take the form of a national identity. This is especially important in non-settler countries where the ethnic majority population is seen as representative for the country as a whole and where it might be initially more difficult for ethnic minority children to identify as nationals (see Munniksma et al., 2015). Indeed, several studies in these non-settler countries suggest that contact with ethnic majority group members can increase national belonging in ethnic minorities (e.g., Kende et al., 2020).

Intergroup Contact theory has been extensively tested in the school context (see Tropp & Prenovost, 2008), and although contact can take negative forms such as discrimination or conflict research, the mere opportunity for contact tends to have a positive effect overall. For this reason, and also because it included classrooms without ethnic majority students, the present study focused on opportunities for contact with out-group peers (rather than the quality of this contact). Just a few contact studies have examined children’s national belonging. Still, research has shown that students with an immigrant background in both primary (Agirdag et al., 2011) and secondary schools (Gharaei et al., 2018) reported a stronger sense of national belonging when they had more ethnic majority classmates, and thus more opportunities for contact with the ethnic majority out-group. Therefore, it was expected that ethnic minority students with a larger share of ethnic majority classmates would have a stronger sense of national belonging.

Whereas school-based research on intergroup contact has almost exclusively focused on the impact of peers (Tropp & Prenovost, 2008), teachers seem to play a role as well (see Thijs et al., 2018). Teachers can be considered representatives of the national educational system, and in many countries most of them have an ethnic majority background (Thijs & Verkuyten, 2012). Therefore, teachers might function as a bridge to the “host” society for students with immigrant backgrounds, and close (i.e., warm and supportive) relationships with them^2^ can be regarded a form of positive intergroup contact which contributes to the development of a common national identity and a sense of national belonging. Indeed, previous research has found a link between teacher support and the national identification of German primary school children over time (Spiegler et al., 2018). Furthermore, a study in the Netherlands found that ethnic minority students who had a closer relationship with their teacher had more positive attitudes towards Dutch people in general. This effect was stronger in classrooms with a smaller share of ethnic Dutch peers (Thijs & Verkuyten, 2012), indicating that contact with a teacher becomes even more important for ethnic minority students who have less opportunities for contact with ethnic majority peers. A comparable finding was obtained among students in Islamic primary schools in the Netherlands – where the school population exclusively consists of Muslim students with mainly immigrant backgrounds. Those students had Muslim classmates only and reported a stronger national belonging if they had a non-Muslim as compared to a Muslim teacher (Thijs et al., 2018). Unlike the earlier Dutch studies, the present study examined national belonging in ethnic minority students from non-Islamic schools. It was expected that a close relationship with their teacher would increase ethnic minority students’ sense of national belonging, especially in classrooms with fewer ethnic majority peers.

### Diversity Norms

Ethnic minority students’ sense of national belonging may also depend on the perceptions of the diversity norms expressed by their teacher and peers. The present study focused on the prescriptive multicultural norms of their teacher as well as the descriptive norms of their classroom peer group. Whereas prescriptive norms refer to expectations of and for behavior (“what should be done or believed”), descriptive norms refer to perceptions of what is ‘normal’ (“what is typically done or believed”) within a social group (Lapinski & Rimal, 2005). Teachers have a formal task to teach students how to deal with diversity (Verkuyten & Thijs, 2013). Given a formal hierarchy between teachers and students, but not among classmates, we focused on prescriptive teacher norms and descriptive peer norms.

#### Classmates’ Norms

Classmates’ descriptive ethnic norms were examined by measuring children’s perceptions of the common ethnic group attitudes in their classroom (“What do most kids in your classroom think about…?”). These perceived norms can function as descriptive norms as they indicate what evaluative responses are “normal” among classmates (see Lapinski & Rimal, 2005). The present study focused on norms concerning the Dutch majority group – which was considered to be most representative for the national group – as well as the ethnic in-group of the minority participants. Depending on the identities of their classmates, these peer norms could have different implications for ethnic minority children’s national belonging. On the one hand, children could adopt their classmates’ norms about the ethnic majority group, and depending on the norm, develop a more positive attitude toward that group themselves. In turn, such a positive attitude could facilitate a common national identity, and thereby increase ethnic minority students’ national belonging. Several theoretical perspectives including social identity development theory (Nesdale, 2004) and socio-cognitive developmental theory (Rutland et al., 2010) claim that children’s out-group attitudes are increasingly governed by peer norms when they get older. Moreover, ethnic minority students have been found to internalize their peers’ attitudes towards the ethnic majority out-group, especially if these peers belong to their own group (De Tezanos-Pinto et al., 2010). Hence, it was expected that a positive peer norm towards the ethnic majority group may increase ethnic minority students’ national belonging, especially in classrooms with a smaller share of ethnic majority peers.

In addition, ethnic minority students could also experience a stronger sense of national belonging if they perceive a positive peer norm about their own ethnic group, because that would indicate that their group is accepted and valued (see Erdal & Strømsø, 2018). Moreover, this effect should be particularly strong if the norm is endorsed by the ethnic majority group, who are most representative for the national group in non-settler countries. Following the *Rejection-Disidentification Hypothesis* (Jasinskaja-Lahti et al., 2009), (feelings of) rejection by the ethnic majority can lead to national disidentification in ethnic minority group members, and this hypothesis has been supported among both adults (e.g., Bobowik et al., 2017) and youth (e.g., Mazzoni et al., 2020). It was therefore expected that a positive perceived norm about *their* in-group would enhance ethnic minority students’ national belonging, especially in classrooms with a larger share of ethnic majority peers because this norm would indicate acceptance by their ethnic majority peers.

#### Teacher’s Norms

Teachers have a formal task to teach students how to deal with diversity and one way of doing so is by prescribing multicultural norms. Research has shown that children’s ethnic out-group attitudes can become more positive when teachers stress that (cultural) differences between groups should be recognized, valued, and seen as a resource (Verkuyten & Thijs, 2013). These prescriptive multicultural teacher norms could have two complementary effects on the national belonging of ethnic minority students. On the one hand, they could increase their positivity toward the ethnic majority out-group (Schachner, 2019). On the other hand, they could also make ethnic minority children feel more welcome and accepted in the country (see Gharaei et al., 2019). As argued above, both effects could stimulate the development of a common national identity. Therefore, it was expected that ethnic minority students’ sense of national belonging is predicted by the multicultural norms expressed by their teachers.

## The Present Study

Ethnic minority children’s sense of national belonging could potentially be fostered in the school context (e.g., Agirdag et al., 2011). Yet, not much research has addressed how the school environment, and what factors in it, can contribute to this. The current cross-sectional study aimed to address this gap in the literature by assessing whether and how the national belonging of ethnic minority preadolescents of Turkish, Moroccan, and Surinamese descent in the Netherlands could be predicted by the opportunity for contact with ethnic majority classmates and the quality of contact with their teachers, as well as the perceived diversity norms of their classmates and teachers. It was expected that ethnic minority students would experience more national belonging in classes with a larger share of ethnic Dutch peers (Hypothesis 1). Moreover, it was hypothesized that ethnic minority students who reported a closer relationship with their teacher, reported a higher sense national belonging (Hypothesis 2a) and that this effect would be stronger in classrooms with a smaller share of ethnic Dutch peers (Hypothesis 2b). Third, it was tested whether ethnic minority students who perceived positive peer norms regarding the ethnic Dutch majority (out-group) experienced stronger feelings of national belonging (Hypothesis 3a) and whether this effect was stronger in classes with a smaller share of ethnic Dutch majority group peers (Hypothesis 3b). Fourth, it was expected that ethnic minority students would experience more national belonging when they perceived a positive peer norm about their in-group among their classmates (indicating more in-group acceptance; Hypothesis 4a) and that this effect would be stronger in classrooms with a larger share of ethnic Dutch students (Hypothesis 4b). Fifth, it was hypothesized that ethnic minority students who perceived a more frequent expression of multicultural norms by their teacher would report more national belonging (Hypothesis 5). Finally, although not the main focus of the present study, national belonging of the ethnic Dutch classmates of the participants was also examined. The underlying assumption was that the national belonging of ethnic minority children would be lower in comparison to that of their ethnic majority peers. This assumption was directly tested, and the current study also explored whether the national belonging of ethnic majority children was predicted by similar factors as that of ethnic minority children.

## Methods

### Procedure

The data were collected in two waves between February and June 2014 as part of a larger project on teacher’s dealing with diversity. In this project, schools were oversampled for ethnic diversity. This was done in two steps. First, in every province of the Netherlands, the largest cities in which at least 10% of the inhabitants had a non-Western immigrant background were selected. Second, in the selected provinces, schools were selected if they had at least 75 students of whom at least 25 were Turkish- or Moroccan-Dutch (for details see Geerlings et al., 2019). Students were recruited through their teachers, and parents of students were informed about the procedures of the study. Passive parental consent was obtained for 96% of students. The study was approved by the institutional review board of the University of Amsterdam in the Netherlands.

Students filled out a paper and pencil questionnaire anonymously in the classroom during regular class hours. This took about 30 min. Most scales were only assessed at one of the two waves to reduce the burden for the participating students. Teacher closeness, classmates’ diversity norms, perceived multicultural teacher norms, and socio-economic status were measured at the first wave. National belonging was only measured at the second wave, which made it impossible to examine changes over time, and therefore the study was cross-sectional in nature. In some cases (13.5%), classes participated with two teachers. In this case, the class was divided in half and each half was assigned to a particular teacher. Students were then asked to answer the teacher-related questions (see Instruments) for that specific teacher.

### Participants

The original sample consisted of 864 primary school students in Grade 4 (27.7%), Grade 5 (36.0%), and Grade 6 (36.3%) and their teachers (*N* = 42). They were from 18 different schools, mostly located in urban areas in the Netherlands, and from 37 classes, with an average of 23 students (*SD* = 4.4) participating students per class. Two schools participated with 5 classes, five schools with 3 classes, four schools with 2 classes, and seven schools with 1 class. This resulted in an average of 2 participating classes per school. Only two participating schools were located in the same city.

Students were classified into ethnic groups based on their self-identification. Students were categorized as ethnic Dutch when they consistently self-identified as Dutch at both waves and when both of their parents were born in the Netherlands. Students were categorized as Turkish, Moroccan, or Surinamese^3^ if they consistently self-identified as such at both waves. When ethnic self-identification at one wave was missing, self-identification at the other wave was used to categorize students into ethnic groups, since self-identification at both waves was highly consistent (84.7%). In addition, to categorize ethnic Dutch students, parents’ country of birth was used as extra an additional criterium. Finally, information on ethnic background was unknown for 29 students (3.4%) due to either inconsistent self-identification (*N* = 27) or missing information at both waves (*N* = 2). These students were excluded from the analyses, but the 27 students with inconsistent self-identification were used in the computation of the proportion of ethnic Dutch students in the classroom (see Instruments).

After this categorization most of the 835 remaining students had an ethnic Dutch background (45.4%), 11.4% of the students self-identified as Turkish, 8.7% as Moroccan, 1.8% as Surinamese, and 32.7% as other than the target groups (e.g., Afghan, Brazilian, Chinese, Nigerian, Romanian, and Syrian). Across the schools, the total percentage of ethnic majority students varied from 6.7% to 87.5%. The total percentage of minority students varied from 12.5% to 100%. The percentage of Moroccan students varied from 1.5% to 65.2%, the percentage of Turkish students ranged from 0.8% to 50%, and the percentage of Surinamese students’ varied from 2.5% to 7.6%. For the final sample, the 273 students whose ethnic background was categorized as *other* were excluded, because there was only information available for Turkish, Moroccan, Surinamese, or Dutch groups regarding classmates’ diversity norms (see Instruments). Moreover, the eight classes with in total 166 ethnic Dutch students were excluded from the analyses because these students were in a class with no Turkish, Moroccan, or Surinamese classmates.

The final sample consisted of 396 students (50.3% female) in 29 classes: Grade 4 (33.3%), Grade 5 (30.8%), and Grade 6 (35.9%). The separate ethnic minority groups (Moroccan, Turkish, and Surinamese students) were combined into one overarching category (*N* = 183) as the three ethnic minority groups were too small (respectively, *N* = 73, *N* = 95, and *N* = 15) to compare each ethnic minority group separately to the ethnic Dutch majority group (*N* = 213). The results of a MANOVA with post-hoc comparisons with Tukey correction showed that the three ethnic minority groups did not significantly differ from each other in background characteristics (i.e., socio-economic status, Grade, gender, and age; *p*-values all >0.05). Students’ mean age was 10.53 years (*SD* = 0.99 years). In some cases, two teachers participated with one class, leading to 33 participating teachers (81.8% female) who all considered themselves as being Dutch (at Wave 1). On average, teachers were 40.76 years old (*SD* = 12.51 years), had 15.14 years of teaching experience (*SD* = 11.70 years), and taught on average 25–32 h per week (range 9 to >32 h) in the class they participated with in the study. To exclude the possibility that the findings were dependent on the number of weekly teaching hours, Teacher FTE was included as a moderator variable.

### Instruments

For all scales in the present study, measurement invariance was assessed to ensure that the scales had a similar underlying factor structure for ethnic minority and ethnic Dutch students (see Data Analyses).

#### National Belonging

National belonging was assessed at the second wave with three items: “I feel at home in the Netherlands”, “I am proud of the Netherlands”, and “I like it in the Netherlands”. These items came from an earlier Dutch study in which the items were formulated as questions rather than statements (Verkuyten et al., 2014). Another item from that study (“Do you ever think the Netherlands is really my country?”) was not included in the questionnaire. It had the lowest corrected item-total correlation there, and it might be difficult to answer for children with immigrant backgrounds. The items were scored on a 5-point Likert scale ranging from 0 (*No!*) to 4 (*Yes!*). The internal consistency of the scale was excellent (α = 0.91).

#### Proportion of Ethnic Dutch Students

In order to compute the proportion of ethnic Dutch students in a classroom, the number of students who were categorized as ethnic Dutch (following the description under participants) in these classes were divided by the total number of students reporting on their ethnic background in the selected classes. A higher percentage indicated that the class had a higher share of students with an ethnic Dutch background. On average, 30.8% (*SD* = 21.5%; range 0%–69%) of the students in a classroom had an ethnic Dutch background.

#### Teacher Closeness

Teacher closeness was assessed at the first wave using the closeness subscale from the Student Perception of Relationship with Teacher Scale (SPRTS; Koomen & Jellesma, 2015). The subscale consisted of six items. Example items are “I feel relaxed with my teacher” and “I think I have a good relationship with my teacher”. Answers were given on a 5-point Likert scale ranging from 0 (*No, absolutely not!*) to 4 (*Yes, absolutely!*). The internal consistency of this scale was good (α = 0.80).

#### Classmates’ Diversity Norms

The perceived norms of the classmates regarding several ethnic groups were assessed at the first wave using a variation of the “Seven Faces” scale (Yee & Brown, 1992). That is to say, students were asked to indicate how *most of their classmates* evaluated various ethnic groups living in the Netherlands, including Dutch, Turkish, Moroccan, and Surinamese people. Answers were given on a 7-point Likert-type scale, ranging from 0 *(big smile/very happy)* to 6 (*big frown/very sad)*. Afterwards, the scale was recoded so a higher score indicated a more positive norm towards the group. Based on these questions two types of perceived group norms were calculated, taking into account the student’s perspective. *Perceived in-group norms* represented the perceived evaluation of a students’ in-group by their classmates. For example, the perceived in-group norm for Turkish students was derived from their perceived evaluation of Turkish people by their classmates, with a higher score indicating a more positive in-group norm. Since the perceived in-group norm was based on one item, no reliability value could be computed. *Perceived out-group norms* represented students’ evaluation of their classmates’ evaluation of out-group members. For ethnic minority group students, this score reflected their evaluation of how their classmates evaluated Dutch people. As this measure was based on only one item for each group, no reliability value could be computed. For ethnic Dutch students, perceived out-group norms were calculated as the mean norm towards ethnic minority group members (i.e., Turkish, Moroccan, and Surinamese people) in general. The internal consistency of the scale was good (α = 0.76). For both groups, a higher score indicated more positive out-group norms.

#### Perceived Multicultural Teacher Norms

Perceived multicultural teacher norms were assessed at the first wave by three items that were successfully used in previous research (Thijs & Verkuyten, 2012): “Does your teachers sometimes tell you to respect all cultures?”, “Does your teacher sometimes tell you not to discriminate?”, and “Does your teacher sometimes tell you that all people from different cultures are equal?”. Questions were answered on a 5-point Likert scale ranging from 0 (*Never*) to 4 (*Very often*). The scale had sufficient internal consistency (α = 0.77).

#### Socio-Economic Status

Students’ socio-economic status (SES) was measured at the first wave using an index scale based on the Family Affluence Scale (FAS; Boyce et al., 2006). Students were asked for the number of cars, computers (including laptops and iPads), bedrooms, and televisions in their household. Answers were given on a scale from 0 (e.g., no car) to 3 (e.g., 3 or more cars). This is a common way to inquire about students’ SES, yet serves only as a proxy as it only involves one aspect of SES, that is family wealth (e.g., Sirin, 2005).

### Data Analyses

#### Measurement Invariance

Prior to the main analyses, it was investigated whether the scales for national belonging, teacher closeness, and perceived multicultural teacher norms were invariant for ethnic minority and ethnic majority students using Mplus (version 8.5; Muthén & Muthén, 2020). Scalar invariance is a prerequisite for comparing group means, as it indicates that the construct actually has the same underlying structure in each group (Van de Schoot et al., 2012). The Alignment Method (Asparouhov & Muthén, 2014), which is a new method to test for measurement invariance, was used. More conventional tests for measurement invariance are often considered too strict, whereas the alignment method allows the parameters in the model to differ slightly from each other in order to still be invariant (Asparouhov & Muthén, 2014). Another advantage of this method is that it can handle a small number of factor indicators and nonnormality of the parameters (Muthén & Asparouhov, 2018).

The alignment method produces an average invariance index which indicates the degree of confidence with which the latent factor means of all constructs in the model can be meaningfully compared across groups. This *R*^*2*^ value ranges from 1, representing perfect scalar invariance to 0, representing full noninvariance. In this case, the average invariance index was relatively high, *R*^2^ = 0.84. This indicates a high degree of confidence with which the two groups on the mean scores of the variables can be compared. For more details on this method, the syntax, and the findings for each separate construct, see the “Detailed description of measurement invariance analyses” (Online Resource).

#### Main Analyses

First, descriptive analyses were performed. Second, in order to examine which factors were predictive of ethnic minority students’ national belonging, a regression model for the sample of ethnic minority students was estimated in Mplus (version 8.5; Muthén & Muthén, 2020), using the factor scores retrieved from the measurement invariance analysis. This model included all predictors and therefore simultaneously tested Hypotheses 1 to 5. The hierarchical structure in the data (i.e., students nested in teachers) was taken into account by using cluster-robust standard errors (i.e., including “type=complex” in the Mplus syntaxes; McNeish et al., 2017). Intraclass correlations were calculated to check the amount of variance at the group level. National belonging served as the dependent variable and the proportion of ethnic Dutch students, teacher closeness, perceived in-group norm, perceived out-group norm, and perceived multicultural teacher norms were entered as predictors. All variables were grand-mean centered before entering them in the model. Moreover, interaction terms for proportion of ethnic Dutch students and teacher closeness, proportion of ethnic Dutch students and perceived in-group norm, and proportion of ethnic Dutch students and perceived out-group norm were included in the model as predictors. All predictors were entered at the same time as no specific expectations were in place regarding the order in which predictors needed to be added. The tested models were saturated and therefore model fit indices could not be compared and are not reported. Standardized beta’s (b*) were used as a measure for effect size. A value of 0.1 corresponds to a weak effect, 0.3 to a moderate effect, and 0.5 to a strong effect (Cohen, 1988). After examining the model for the ethnic minority students, a similar regression model was tested for the ethnic Dutch students in order to explore whether their national belonging was predicted by similar factors.

Beforehand, assumptions for t-tests, correlational and regression analyses were checked. All assumptions were met except for the assumption of normality for national belonging of the ethnic Dutch students, and perceived in-group norms of both groups. To account for this non-normality, MLR estimation (maximum likelihood estimation with robust standard errors) was employed in the Mplus analyses. Missing value analysis indicated that national belonging had the most missing values (8.8%). For all other variables in the present study, less than 5% of the data was missing, or no data was missing at all (range 0.0% - 3.8%). Missing values were all located on the individual level and no classes were excluded due to missing data. Moreover, Little’s MCAR test was not significant, χ2 = 116.63, DF = 100, *p* = 0.122, suggesting that data was missing completely at random. As such, in addition to the MLR estimation, which uses full information maximum likelihood to handle missing data, the cases with missing values on the dependent variable were excluded from analysis.

## Results

### Preliminary Analyses

Descriptive statistics for the total sample, as well as for the ethnic minority students and ethnic Dutch students separately are depicted in Table 1. The Intraclass Correlations *(ICCs)* indicated that for all variables, most variance was located at the student level as compared to the teacher level, demonstrating substantial variation between students with the same teacher. Compared to ethnic Dutch students, ethnic minority students reported less national belonging, less closeness with their teacher, and had a lower socio-economic status. Values of Cohen’s *d* suggest that these differences can be interpreted as large, small, and medium respectively. Moreover, compared to their ethnic Dutch classmates, ethnic minority group students were older and reported more positive peer norms about their out-group, and a more frequent expression of multicultural norms by their teacher. These differences can all be interpreted as small. Both groups reported similar in-group norms. Since both groups did not differ in grade and gender, these variables were not included in further analyses.

**Table 1:** Descriptive statistics of within-level variables for the total group, and separately for ethnic minority and ethnic Dutch students

	Total group			ICC	Ethnic minority students			Ethnic Dutch students			t-Test	*d*
	*N*	*M*	*SD*		*N*	*M*	*SD*	*N*	*M*	*SD*		
National belonging	361	3.15	0.95	0.25	168	2.68	1.05	193	3.57	0.61	−9.60***	−1.05
Teacher closeness	384	2.74	0.83	0.18	172	2.58	0.92	212	2.87	0.73	−3.42**	−0.36
Perceived in-group norms	383	5.33	1.12	0.12	171	5.22	1.17	212	5.41	1.07	−1.64	−0.17
Perceived out-group norms	381	4.29	1.48	0.13	169	4.49	1.79	212	4.13	1.17	2.21*	0.24
Perceived multicultural teacher norms	385	2.41	1.02	0.23	172	2.57	1.12	213	2.28	0.93	2.70**	0.28
SES	384	2.17	0.41	0.08	173	2.06	0.44	211	2.26	0.37	−4.67***	−0.49
Age	385	10.53	0.99	0.61	172	10.67	0.98	213	10.41	0.98	2.64**	0.27
Grade	396	7.03	0.83	0.91	183	7.05	0.78	213	7.00	0.87	0.66	0.07
Gender	395	0.50	0.50	0.03	182	0.47	0.50	213	0.54	0.50	−1.35	−0.14

*p < 0.05 level (2-tailed); **p < 0.01 level (2-tailed); ***p < 0.001 level (2-tailed)

Table 2 shows the correlations between the variables for each group separately. For the ethnic minority group students, national belonging was positively associated with teacher closeness, perceived out-group norms, and perceived multicultural teacher norms. Interestingly, ethnic minority students’ perceived out-group norms were positively associated with the percentage of ethnic Dutch students in the classroom and the perceived multicultural teacher norms.

**Table 2:** Correlations of variables in the present study for ethnic minority and ethnic Dutch students separately

	1.	2.	3.	4.	5.	6.	7.	8.
1. National belonging	–	0.05	0.18**	0.21**	0.15*	0.08	0.09	0.10
2. % Ethnic Dutch peers	0.18	–	0.16*	0.43***	−0.07	−0.18*	0.25***	−0.07
3. Teacher closeness	0.25***	0.18*	–	0.21*	0.16*	0.12	0.11	0.06
4. Perceived in-group norms	0.03	−0.09	0.03	–	0.04	−0.05	0.21**	0.03
5. Perceived out-group norms	0.29**	0.32***	0.41***	−0.00	–	0.24**	−0.13	0.08
6. Perceived multicultural teacher norms	0.19*	−0.13	0.34***	−0.09	0.19*	–	−0.11	0.33***
7. SES	−0.00	−0.09	−0.17*	0.04	−0.14	0.01	–	−0.02
8. Age	−0.01	−0.18	−0.04	0.05	−0.14*	0.13	0.15	–

Correlations below the diagonal refer to the ethnic minority students and above the diagonal to the ethnic Dutch students

*p < 0.05 level (2-tailed); **p < 0.01 level (2-tailed); ***p < 0.001 level (2-tailed)

For ethnic Dutch students, national belonging was positively associated with teacher closeness, perceived in-group norms, and perceived out-group norms (see Table 2). Interestingly, ethnic Dutch students’ perceived out-group norms were positively associated with perceived multicultural teacher norms.

### Predicting National Belonging

Prior to testing the hypotheses, we examined which covariates needed to be taken into account. The correlations between national belonging and the covariates student age and SES were non-significant for both groups (see Table 2). In addition, it was tested whether student age and SES predicted national belonging in addition to students’ ethnicity. The results show that only students’ ethnicity significantly predicted national belonging (b* = −0.51, *p* < 0.001). It was therefore decided not to include student age and SES in the main analyses as covariates.

#### Ethnic Minority Group Students

**Fig. 1:**
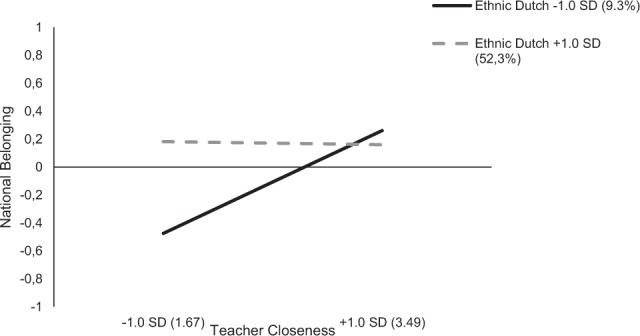
Interaction effect of teacher closeness and percentage ethnic Dutch peers on ethnic minority students’ national belonging (*N* = 169)

To test Hypothesis 1 to 5, a multiple linear regression, with all predictors included, was estimated to examine ethnic minority students’ national belonging. The results are presented in Table 3. In line with Hypothesis 1, ethnic minority group students’ national belonging was higher when they were in classrooms with a larger share of ethnic Dutch peers (b* = 0.21, *p* = 0.017, indicating a weak to moderate effect; Cohen, 1988). Moreover, support for Hypothesis 2a was found, as ethnic minority students’ national belonging was higher when they experienced a more positive relationship with their teacher (b* = 0.19, *p* = 0.024, indicating a weak to moderate effect). Furthermore, in line with Hypothesis 2b, the effect of teacher closeness on ethnic minority students’ national belonging was stronger in classes with fewer ethnic Dutch peers (b* = −0.14, *p* = 0.017, indicating a weak effect). Simple slope analyses showed that the effect of teacher closeness was positive in classes with few ethnic Dutch students (1 *SD* < *M*; *b* = 0.37, *p* < 0.001) but not so in classes with many ethnic Dutch students (1 *SD* > *M*; *b* = −0.01, *p* = 0.922). This interaction is shown in Fig. 1. The other hypotheses (Hypotheses 3a-5), stating that perceived in-group and out-group norms of classmates, their interactions with proportion ethnic Dutch, and the perceived multicultural teacher norms were predictive of ethnic minority students’ national belonging, were not confirmed. In addition, the interactions with teachers’ FTE were non-significant, indicating that the significant effect of teacher closeness and the non-significant effect of multicultural teacher norms were not dependent of the number of weekly teaching hours.

**Table 3:** Unstandardized and standardized estimates for the model predicting ethnic minority students’ national belonging (*N* = 169)

	Model 1			Model 2		
	*B*	*SE*	b*	*B*	*SE*	b*
% Ethnic Dutch peers	1.06*	0.45	0.21*	1.11*	0.46	0.22*
Teacher closeness	0.18*	0.08	0.19*	0.21**	0.08	0.21*
Perceived in-group norms	0.03	0.04	0.04	–	–	–
Perceived out-group norms	0.05	0.04	0.09	–	–	–
Preference towards ethnic Dutch people	–	–	–	0.02	0.02	0.05
Perceived multicultural teacher norms	0.12	0.09	0.12	0.12	0.09	0.12
% Ethnic Dutch peers × Teacher closeness	−0.89*	0.37	−0.14*	−0.89*	0.38	−0.14*
% Ethnic Dutch peers × Perceived in-group norms	0.33	0.22	0.08	–	–	–
% Ethnic Dutch peers × Perceived out-group norms	−0.31	0.22	−0.10	–	–	–
% Ethnic Dutch peers × Perceived Preference towards ethnic Dutch	–	–	–	−0.35**	0.13	−0.15**
Teacher FTE	0.09	0.08	0.10	0.09	0.08	0.10
Teacher FTE × Teacher closeness	−0.02	0.09	−0.02	−0.03	0.08	−0.03
Teacher FTE × Perceived multicultural teacher norms	−0.02	0.10	−0.02	−0.02	0.10	−0.02
Explained variance (R^2^)			0.23**			0.23**

*p < 0.05 level (2-tailed); **p < 0.01 level (2-tailed); ***p < 0.001 level (2-tailed)

Since neither the in-group nor out-group norms of classmates were predictive of ethnic minority students’ national belonging separately, the possibility that classmates’ relative preference towards ethnic Dutch people (versus their in-group), predicted ethnic minority students’ national belonging was investigated. To this aim the difference between the perceived norm towards the Dutch out-group and the perceived in-group norm were calculated. On average, this perceived preference was weak but there was also no strong preference for the in-group (*M* = −0.75; *SD* = 2.14). The perceived preference towards ethnic Dutch people was entered in the previous regression model, replacing the predictors ‘Perceived in- and out-group norms’. Moreover, an interaction term for this perceived preference with the percentage of ethnic Dutch students was entered in the model, replacing the interaction terms for the perceived in-group and out-group predictors.

**Fig. 2:**
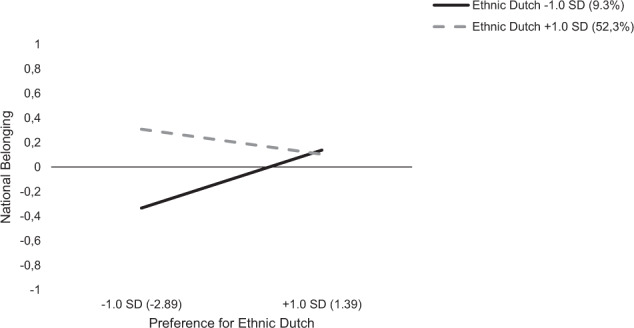
Interaction effect of perceived bias towards ethnic Dutch people and percentage ethnic Dutch peers on ethnic minority students’ national belonging (*N* = 169)

The results for the ethnic minority group (Table 3, Model 2), indicates that the main effect of preference for ethnic Dutch people was not significant, but the interaction between preference and classroom composition was significantly associated with ethnic minority students’ national belonging. That is, ethnic minority students’ national belonging was negatively predicted by the interaction between the percentage of ethnic Dutch peers in the classroom and their perceived preference towards ethnic Dutch people (b* = −0.15, *p* = 0.008, indicating a weak effect). Simple slope analyses showed that the effect of perceived preference was positive in classes with few ethnic Dutch students (1 *SD* < *M*; *b* = 0.24, *p* = 0.003) but not so in classes with many ethnic Dutch students (1 *SD* > *M*; *b* = −0.10, *p* = 0.228). Figure 2 further illustrates this interaction. It appeared that ethnic minority students’ national belonging was the lowest if they had few ethnic Dutch classmates, and if their classmates were perceived to prefer students’ in-groups over ethnic Dutch people.

#### Ethnic Dutch Students

A similar multiple linear regression, with all parameters included, was estimated to explore which factors were predictive of ethnic Dutch students’ national belonging. The results, depicted in Table 4, indicated that ethnic Dutch students experienced more national belonging when they had a closer relationship with their teacher (b* = 0.24, *p* < 0.001, indicating a weak to moderate effect) and when they perceived their classmates as having more positive in-group norms (b* = 0.30, *p* = 0.007, indicating a moderate effect). The other predictors and interactions included in the model were not significantly associated with ethnic Dutch students’ national belonging.

**Table 4:** Unstandardized and standardized estimates for the model predicting ethnic Dutch students’ national belonging (*N* = 212)

	Model 1			Model 2		
	*B*	*SE*	b*	*B*	*SE*	b*
% Ethnic Dutch peers	−0.12	0.20	−0.04	−0.07	0.20	0.02
Teacher closeness	0.17**	0.05	0.24***	0.21***	0.05	0.29***
Perceived in-group norms	0.15**	0.05	0.30**	–	–	–
Perceived out-group norms	0.02	0.04	0.05	–	–	–
Preference towards ethnic Dutch people	–	–	–	0.03	0.04	0.07
Perceived multicultural teacher norms	0.04	0.05	0.06	0.06	0.06	0.08
% Ethnic Dutch peers × Teacher closeness	0.33	0.40	0.09	0.25	0.38	0.07
% Ethnic Dutch peers × Perceived in-group norms	0.28	0.20	0.14	–	–	–
% Ethnic Dutch peers × Perceived out-group norms	−0.09	0.14	−0.04	–	–	–
% Ethnic Dutch peers × Perceived Preference towards ethnic Dutch	–	–	–	0.00	0.13	0.00
Teacher FTE	0.04	0.03	−0.08	−0.03	0.03	−0.06
Teacher FTE × Teacher closeness	0.07	0.04	0.11	0.06	0.04	0.08
Teacher FTE × Perceived multicultural teacher norms	0.03	0.05	0.04	0.04	0.04	0.06
Explained variance (R^2^)			0.14***			0.10**

*p < 0.05 level (2-tailed); **p < 0.01 level (2-tailed); ***p < 0.001 level (2-tailed)

The present study also investigated the effect of preference for ethnic Dutch people on ethnic Dutch students’ national belonging. Again a positive score indicated a preference of classmates in favor of ethnic Dutch students. On average, this perceived preference was weak but there was also no strong preference for the in-group (*M* = 1.28; *SD* = 1.55). The results, as presented in Table 4 under Model 2, indicated that the main effect of perceived preference nor its interaction with classroom composition were significant predictors of ethnic Dutch students’ national belonging.

Finally, to examine whether the associations between national belonging and the predictors differed between ethnic minority and ethnic Dutch students, *z-*scores were calculated for the differences between the unstandardized regression coefficients of the predictor pairs using the following equation (e.g., Paternoster et al., 1998). A significant difference between the regression coefficients was indicated by a *z-*score above 1.96.

$$Z = \frac{{b_{{\mathrm{predictor}}\,{\mathrm{ethnic}}\,{\mathrm{minority}}\,{\mathrm{students}}}\;-b_{{\mathrm{predictor}}\,{\mathrm{ethnic}}\,{\mathrm{Dutch}}\,{\mathrm{students}}}}}{{\sqrt {\left( {{\mathrm{SE}}_{{\mathrm{predictor}}\,{\mathrm{ethnic}}\,{\mathrm{minority}}\,{\mathrm{students}}}} \right)^2 + \left( {{\mathrm{SE}}_{{\mathrm{predictor}}\,{\mathrm{ethnic}}\,{\mathrm{Dutch}}\,{\mathrm{students}}}} \right)^2} }}$$

The results for Model 1 indicated that the effect of the percentage of ethnic Dutch students in the classroom (*Z* = 2.41; *p* = 0.016) as well as the interaction of percentage ethnic Dutch with teacher closeness (*Z* = −2.40*; p* = 0.016), which were only significant predictors for ethnic minority students, differed between the two groups. The other effects did not significantly differ between groups (*p-*values > 0.05).

Finally, for Model 2, the *z*-scores indicated that the effect of the percentage of ethnic Dutch students in the classroom *(Z* = 2.11; *p* = 0.035), the interaction of percentage ethnic Dutch and teacher closeness (*Z* = −2.12; *p* = 0.034), as well as the interaction of percentage ethnic Dutch and perceived preference for ethnic Dutch people (*Z* = −1.98; *p* = 0.048), which were only significant predictors for ethnic minority students, differed between the two groups. The other effects did not significantly differ between groups (*p-*values > 0.05).

### Robustness Checks

Various robustness checks were conducted. First of all, due to the fact that in some cases two teachers participated with one class and students answered questions about a specific teacher, students were nested in teachers as well as in classes. In the main analyses, the nesting in teachers was accounted for as it was not possible to simultaneously account for the nesting in classes and teachers. To check the robustness of the findings in the present study, it was examined if the results of the models would differ when students were examined as nested in classes. The results of these analyses were similar to those when students were nested in teachers, both for the ethnic minority group students and for the ethnic Dutch students.

Moreover, students from ethnic minority groups were grouped into one overarching category. However, a MANOVA with post-hoc comparisons with Tukey correction, showed that Turkish and Moroccan students significantly differ in their national belonging (*p* < 0.05), suggesting that classroom factors may work differently for students in different ethnic minority groups. Therefore, it was checked if and how the results would differ if the models were tested separately for different ethnic minority groups. This was only done for the Turkish (*N* = 95) and Moroccan (*N* = 73) students, as the sample of Surinamese students was too small (*N* = 15) to include separately and this group did not significantly differ from the other groups in any of the variables of the present study (*p*-values all >0.05). The results show that, partly due to the lower sample sizes, some paths that were significant for the combined group did not reach significance for the separate groups. Yet, overall, the results followed a similar pattern and findings were mostly in similar directions as the results for the ethnic minority groups combined. For detailed results of the robustness checks, see the “Detailed results of robustness checks” (Online Resource).

## Discussion

Ethnic minority group students can experience a low sense of belonging to the countries they live in (e.g., Fleischmann & Phalet, 2018) and this could hamper their psychological well-being and social adjustment (Wu et al., 2018). Even though schools are important places for the development and stimulation of national belonging in ethnic minority youngsters (Spiegler et al., 2018), few studies have examined how the school environment can contribute to ethnic minority children’s sense of national belonging (e.g., Agirdag et al., 2011). Therefore, the present study investigated whether and how interethnic contact and perceived diversity norms of the teacher and classmates were associated with ethnic minority students’ sense of national belonging. The results indicate that ethnic minority students had a considerably lower sense of national belonging than their ethnic majority peers, which might be due to their current societal status in the Netherlands. However, this sense of belonging was fostered by contact opportunities with ethnic Dutch students and a close relationship with the teacher. And although the perceived diversity norms expressed by their teacher and classmates had no effects, the relative peer norms (perceived attitudes towards ethnic Dutch people versus the in-group) proved to be relevant, especially in classes with fewer ethnic Dutch students.

Based on *Intergroup Contact Theory* (Allport, 1954) and the *Common In-Group Identity Model* (Dovidio et al., 2007), it was expected that the national belonging of ethnic minority students would depend on the share of ethnic Dutch students in their classroom (contact opportunity) and the relational closeness (contact quality) with their teachers. The findings supported both of these hypotheses. Consistent with earlier research (e.g., Agirdag et al., 2011), it was indeed found that having more ethnic Dutch classmates and a closer relationship with their teachers contributed to a stronger sense of national belonging. Moreover, the relationship with the teacher was even more important in classrooms with fewer ethnic Dutch children. These findings suggest that a mixed school population in terms of students’ ethnic background can help ethnic minority students to feel at home in their host society. However, due to residential segregation (Bolt et al., 2010), not all schools have such a mixed ethnic population. The findings seem to suggest that in these cases, high-quality relationships with teachers, can compensate for a lack of contact opportunities with ethnic majority classmates. For future research it would be interesting to examine whether this effect is due to the ethnic majority background of the teacher (as most likely all or most teachers in the sample had an ethnic Dutch background) or whether this also holds for teachers with an ethnic minority background. It may be that ethnic majority teachers are a source of interethnic contact for ethnic minority students (Thijs & Verkuyten, 2012), or alternatively, that the teacher, regardless of their background, is considered to be a representative of the “host” society, and thus important for ethnic minority children’s sense of national belonging.

The current study also examined the effect of perceived diversity norms of classmates (i.e., perceived in- and out-group norms) and perceived multicultural teacher norms on ethnic minority students’ national belonging. In line with social identity development theory (Nesdale, 2004) and socio-cognitive developmental theory (Rutland et al., 2010) it was expected that ethnic minority children who experienced a positive peer norm towards the ethnic majority group internalized this group norm and in turn experienced more national belonging, especially if they were in classes with fewer ethnic Dutch peers. In addition, based on the *Rejection-Disidentification hypothesis* (Jasinskaja-Lahti et al., 2009) it was expected that perceived norms of classmates towards their in-group would be associated with ethnic minority students’ national belonging. These expectations were not directly confirmed as neither the perceived in-group norms nor the perceived out-group norms of classmates were associated with ethnic minority students’ national belonging. Still, the results of additional analyses did provide some support for the internalization of norms about the ethnic majority group and the Rejection-Disidentification hypothesis. That is to say, a perceived peer norm in favor of ethnic Dutch people had a positive effect in classes with fewer ethnic Dutch peers, suggesting that children adopted or internalized this norm, but no effect in classrooms with more ethnic Dutch peers, suggesting that they felt rejected by it. Ethnic minority students’ national belonging thus seems to depend more on the relative, than on the absolute norms expressed about different groups. Another interpretation of this finding is that although contact with ethnic majority group members positively predicted ethnic minority students’ national belonging, this contact alone was not enough. They also needed to perceive a relatively positive attitude among their classmates towards their group.

Moreover, based on previous studies (e.g., Gharaei et al., 2019), it was expected that the perceived multicultural teacher norms would contribute to ethnic minority students’ sense of national belonging. However, even though teachers’ perceived multicultural norms were associated with a stronger perception of positive out-group norms among ethnic majority students, suggesting that they could create more welcoming attitudes towards ethnic minority students, they were not found to be associated with ethnic minority students’ national belonging. An explanation for this could be that although teachers might promote a more open, inclusive, and safe environment for all students through the expression of their multicultural norms, the expression of these norms may also, unintentionally, highlight differences between ethnic minority and ethnic majority students. This could make ethnic minority students more aware of for example discriminating practices towards their group and, in turn, hamper their sense of national belonging (Verkuyten & Thijs, 2013).

Although not the main focus of the present study, the present study also explored ethnic Dutch students’ national belonging. Even though ethnic majority students reported a higher sense of national belonging than ethnic minority students, this belonging was also dependent on classroom factors. More specifically, it was found that a close relationship with the teacher and perceiving a positive in-group norms of classmates, but not the other factors, were associated with ethnic Dutch students’ sense of national belonging. Contrary to the findings for ethnic minority students, the percentage of ethnic Dutch classmates was not associated with ethnic Dutch students’ national belonging. This suggests that ethnic diversity in classes does not come at the expense of ethnic majority group students’ national belonging. Furthermore, the expression of multicultural norms by the teacher was not associated with ethnic Dutch students’ national belonging, indicating that multicultural education or learning about different groups and cultures did neither harm nor encourage ethnic Dutch students’ sense of national belonging. Thus, an ethnically mixed classroom where students have the opportunity to get to know students from other ethnic backgrounds does not seem problematic for ethnic Dutch students and appears to be beneficial for ethnic minority group students in terms of their sense of national belonging.

### Limitations and Future Directions

In evaluating the present study, some limitations should be addressed. First, student reports were used to measure the perceived multicultural norms of their teacher and the perceived diversity norms of their classmates. It remains unclear to what extent these perceived norms reflect the actual norms endorsed by teachers and classmates. Nevertheless, these perceived norms might be more important than the actual norms because ultimately norms affect people via their subjective perceptions of them. Second, although the present study included data from two waves, it is not possible to make causal claims regarding the prediction of national belonging. It is not possible to completely rule out the possibility that ethnic minority group students’ sense of national belonging may affect (the quality of) their interethnic contact and their perceptions of the norms of others, but it is highly unlikely that it affects ethnic classroom composition. Nevertheless, future research should use longitudinal data to corroborate the findings. Third, the minority groups were combined into one overarching category due to small sample sizes in each group. Combining the groups into one category did not seem to have much influence on the results, as indicated by the robustness checks. Still, future research could aim to investigate these effects with larger sample sizes for each minority group in order to further examine if classroom factors have different effects on the national belonging of students from different minority groups. Fourth, since schools were oversampled for ethnic diversity, the external validity of the present results is limited to ethnically diverse classrooms. Future research should therefore examine if similar results might be found in more homogeneous contexts or that findings are indeed limited to ethnically diverse schools. Fifth, although the present study focused on classroom factors as predictors of national belonging, other contexts are potentially relevant as well, such as students’ families and neighborhoods. Studying those contexts was beyond the scope of this paper, but future research could also include parental diversity norms or neighborhood characteristics to examine the relative contribution of the school context to ethnic minority (and ethnic majority) students’ national belonging. Finally, whereas students’ ethnicity was measured by asking them about their ethnic backgrounds (and the birth countries of their parents), teachers were asked whether they considered themselves to be Dutch. It could be that teachers interpreted “Dutch” in the national rather than the ethnic sense, and that some of them had ethnic minority backgrounds themselves as well. However, in the teacher questionnaire used to measure teachers’ Dutch identification, the identification question was directly preceded by questions about friends of different backgrounds (including Dutch, Moroccan, Turkish and Surinamese), which makes a national interpretation of “Dutch” unlikely. Moreover, the majority of Dutch teachers are ethnic Dutch as 90% of the teachers in primary school has an ethnic Dutch background (Centraal Bureau voor de Statistiek, 2018).

The results from the present study offer some other interesting lines for future research. First, national belonging of ethnic minority (as compared to ethnic majority) group children was examined. However, following Berry’s et al. (2006) acculturation model, ethnic minority group members can combine a sense of national belonging with a sense of belonging to their ethnic group, a combination referred to as integration. Future research could examine ethnic minority students’ national belonging in relation to their ethnic belonging and study the degree to which they are integrated. Second, national belonging is not the only pathway through which ethnic minority students’ academic attainment or well-being can be promoted. For example, a recent review has shown that a stronger sense of ethnic belonging/ethnic identity and positive attitudes toward school are also vital to ethnic minority students’ educational attainment (Marks & Garcia Coll, 2018). Moreover, a stronger sense of ethnic identity predicts well-being and this effect tends to be stronger for adolescents and young adults compared to middle aged people (Smith & Sylva, 2011). Future research, should therefore include additional outcomes, such as ethnic identity, to shed a light on other resilient pathways that can contribute to minority students’ successful development. Third, schools were sampled in different areas of the Netherlands. Although this was not possible in the present study, future research could also examine regional differences in national belonging. Fourth, multicultural norms can be transmitted through the explicit curriculum, as schools have the task to provide civic education to their students (Council of Europe, 2016), but also through a more implicit/hidden curriculum. This hidden curriculum is “composed of unstated norms, values, and beliefs embedded in and transmitted to the students through underlying rules that structure the routines and social relationships” within the classroom and can be even more powerful than that what is explicitly taught (Giroux, 2001, p. 47). For example, when teachers stress the importance of having respect for members of different groups, but do not practice this themselves, this implicitly teaches students that the explict teacher norms are not relevant. Future research could disentangle the effects of multicultural norms expressed through the explicit and implicit curriculum. Fifth, with respect to peers, contact opportunity rather than actual contact or contact quality was examined. The presence of ethnic majority group students does not have to mean positive contact per se. More recently, studies have started to examine the effects of negative contact between groups and shown that negative contact has more (negative) impact than positive contact (Graf et al., 2014). For future research it could therefore be worthwhile to examine both positive and negative contact and to distinguish their unique effects on national belonging. Finally, future research on minority students’ sense of national belonging might also include different age groups. The present study focused on preadolescence, as this was considered an appropriate period for stimulating a positive national identity. However, children’s national identification continues to develop throughout adolescence (Barrett, 2007), and it is important to examine the role of the school environment in that development. As secondary school students tend to have several teachers, it can be speculated that interpersonal relations with teachers are probably less important for the national belonging of minority adolescents. However the perceived norms of classmates might still be relevant as peers tend to be most influential around age 14 (Berndt, 1979).

### Practical Implications

The findings of the current study have several practical implications. The finding that ethnic minority students’ national belonging is higher when they have the opportunity to come in contact with peers from the ethnic majority group implies that schools should aim for a mixed student population. Specifically, this means that national or local policies may need to be implemented aimed at countering school segregation. Prior research in the Netherlands (Peters & Walraven, 2011) has suggested that parent-focused policies – such as facilitating parental initiatives to reduce segregation, providing parents with information and advice, or limiting parents’ school choice – could be effective ways of countering school segregation. In addition, the finding that ethnic minority students’ sense of national belonging was lowest in classes with a high share of ethnic minority students where classmates were seen to favor their minority in-group not only supports the idea of implementing policies to decrease school segregation. It also suggests that teachers of segregated classes with mostly ethnic minority students need to be aware that their students may develop a bias against the ethnic majority group. Prior research suggests that such a bias may be counteracted by interventions that, for example, focus on increasing students’ empathy and perspective taking (Beelmann & Heinemann, 2014). Moreover, the finding that ethnic minority students experience less national belonging in classes with more ethnic minority group peers, especially when they have a less close relationship with their teacher, further highlights the importance of tackling school segregation, but also calls upon teachers to establish a warm and positive relationship with their students. Research has shown that teachers can establish positive relationships by acting friendly and supportive but at the same time provide structure to their students (Pennings et al., 2018).

## Conclusion

Previous research in various countries suggests that people with ethnic minority or immigrant backgrounds can experience a low sense of belonging to the countries they live in, or even feel alienated from these countries, due to experiences of discrimination and marginalization (e.g., Verkuyten & Martinovic, 2012). Few studies examined how ethnic minority students’ sense of national belonging can be fostered at school. The present study examined classroom factors that predicted ethnic minority (versus ethnic majority) students’ national belonging. Its findings suggested that a close relationship with the teacher and the presence of ethnic Dutch classmates, may help those students to feel more at home in their “host” society. Moreover, ethnic minority students who perceived a relatively positive norm towards their group reported a higher sense of belonging, but only in classrooms with many ethnic Dutch classmates. In segregated classrooms, the effect of this perceived norm hampered their national belonging. Thus, in order to make ethnic minority students feel more at home in their country, schools should not only aim for a mixed student population, but also consider the norms in the classroom, and the possibility that those norms could have widely different effects depending on the classroom’s ethnic composition. Moreover, the results show that the school environment can foster the national belonging of preadolescent ethnic minority group students, which in turn could help prevent these students from alienating from the “host” society at a later age. It is important that all children feel at home in the country they live in, and hopefully, the findings of the present study can be used to achieve this.

## Supplementary information

Supplementary Information 1

Supplementary Information 2

## Footnotes

1 The term ‘ethnic’ rather than ‘native’ Dutch is used as most people of Turkish, Moroccan, and Surinamese descent in the Netherlands were born in the country.

2 As Dutch primary school students usually have no more than one or two teacher(s) throughout the school year, this gives them even more opportunity to form close relationships with them.

3 Students who identified as Hindu were included in the Surinamese group, but only if at least one of their parents was born in Suriname, as Hinduism is one of the majority religions in Suriname.
