# Supplementary material for: Classroom Predictors of National Belonging: The Role of Interethnic Contact and Teachers’ and Classmates’ Diversity Norms
Source: J Youth Adolesc. 2021 Apr 7;50(8):1709–25. doi: 10.1007/s10964-021-01430-2 (PMC8270874; doi:10.1007/s10964-021-01430-2)
Supplement: Supplementary file 1 — Supplementary Information 1 [file 10964_2021_1430_MOESM1_ESM.pdf]

Supplementary Information

Classroom Predictors of National Belonging: The Role of Interethnic Contact and Teachers' and Classmates' Diversity Norms

Journal of Youth and Adolescence

Contents:

Part One: Results regarding nesting in classes

Part Two: Results regarding Turkish and Moroccan students separately

Part One

Results Regarding Nesting in Classes

In the main analysis, students were nested in teachers, and in some cases two teachers participated with one class with students answering questions about one of them. The robustness of the findings was checked by examining whether the results of the models would differ when examining students as nested in classes. Detailed results can be found below. For both groups of students and both models results were similar to those reported in the main analyses, indicating that the way students were clustered did not substantially affect our results.

Ethnic Minority Group Students

First, minority students' national belonging was examined using a multiple linear regression with all predictors included. The results are presented in Table 1. In line with Hypothesis 1, ethnic minority group students' national belonging was higher when they were in classrooms with a larger share of ethnic Dutch peers ($b^* = 0.21$, $p = .021$,). Moreover, Hypothesis 2a was supported, as ethnic minority students' national belonging was higher when they experienced a more positive relationship with their teacher ($b^* = 0.19$, $p = .028$). Furthermore, in line with Hypothesis 2b, the effect of teacher closeness on ethnic minority students' national belonging was stronger in classes with fewer ethnic Dutch peers ($b^* = -0.14$, $p = .017$). Simple slope analyses showed that the effect of teacher closeness was positive in classes with few ethnic Dutch students (1 $SD < M$; $b = 0.37$, $p < .001$) but not so in classes with many ethnic Dutch students (1 $SD > M$; $b = -0.01$, $p = .921$). This interaction is shown in Figure 1. The other hypotheses (Hypotheses 3a-5), stating that perceived in-group and out-group norms of classmates, their interactions with proportion ethnic Dutch, and the perceived multicultural teacher norms were predictive of ethnic minority students' national belonging, were not supported. In addition, the interactions with teachers' FTE were non-significant, indicating that the significant effect of teacher closeness and the non-significant effect of multicultural teacher norms were not dependent of the number of weekly teaching hours.

Table 1

Unstandardized and Standardized Estimates for the Model Predicting Ethnic Minority Students' National Belonging (N=169)

	Model 1			Model 2		
	B	*SE*	*b**	*B*	*SE*	*b**
% Ethnic Dutch peers	1.06*	0.47	0.21*	1.11*	0.46	0.22*
Teacher closeness	0.18*	0.08	0.19*	0.21**	0.08	0.21*
Perceived in-group norms	0.03	0.04	0.04	-	-	-
Perceived out-group norms	0.05	0.04	0.09	-	-	-
Preference towards ethnic Dutch people	-	-	-	0.02	0.02	0.05
Perceived multicultural teacher norms	0.12	0.10	0.12	0.12	0.09	0.12
% Ethnic Dutch peers x Teacher closeness	-0.89*	0.36	-0.14*	-0.89*	0.35	-0.14*
% Ethnic Dutch peers x Perceived in-group norms	0.33	0.21	0.08	-	-	-
% Ethnic Dutch peers x Perceived out-group norms	-0.31	0.25	-0.10	-	-	-
% Ethnic Dutch peers x Perceived Preference towards ethnic Dutch	-	-	-	-0.35*	0.14	-0.15**
Teacher FTE	0.09	0.07	0.10	0.09	0.07	0.10
Teacher FTE x Teacher closeness	-0.02	0.09	-0.02	-0.03	0.09	-0.03
Teacher FTE x Perceived multicultural teacher norms	-0.02	0.10	-0.02	-0.02	0.09	-0.02
Explained variance (R^2)			0.23**			0.23**

Note. * $p < .05$ level (2-tailed). ** $p < .01$ level (2-tailed). *** $p < .001$ level (2-tailed).

Figure 1

Interaction Effect of Teacher Closeness and Percentage Ethnic Dutch Peers on Ethnic Minority Students' National Belonging (N = 169)

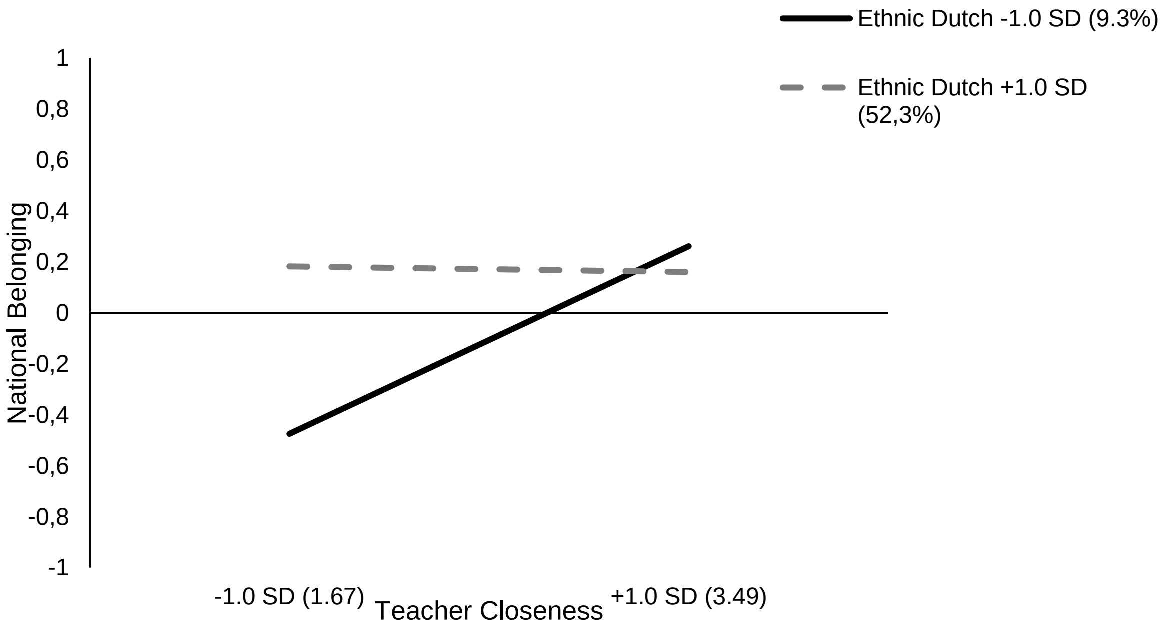

It was also examined if the nesting of students in classes yielded different outcomes for the model including students' perceived preference for ethnic Dutch people (versus their in-group). The results are displayed in Table 1, Model 2 and indicated that the perceived preference for ethnic Dutch people had no main effect, but that the interaction between preference and classroom composition was significantly associated with ethnic minority students' national belonging. That is, ethnic minority students' national belonging was negatively predicted by the interaction between the percentage of ethnic Dutch peers in the classroom and their preference towards ethnic Dutch people (b^* = -.15, p = .016). Simple slope analyses showed that the effect of perceived preference was positive in classes with few ethnic Dutch students (1 $SD < M$; b = 0.24, p = .008) but not so in classes with many ethnic Dutch students (1 $SD > M$; b = -0.10, p = .222). Figure 2 further illustrates this interaction. It appeared that ethnic minority students' national belonging was lowest if they had few ethnic Dutch classmates, and if their classmates were perceived to prefer students' in-groups over ethnic Dutch.

Figure 2

Interaction Effect of Perceived Bias Towards Ethnic Dutch People and Percentage Ethnic Dutch Peers on Ethnic Minority Students' National Belonging (N=169)

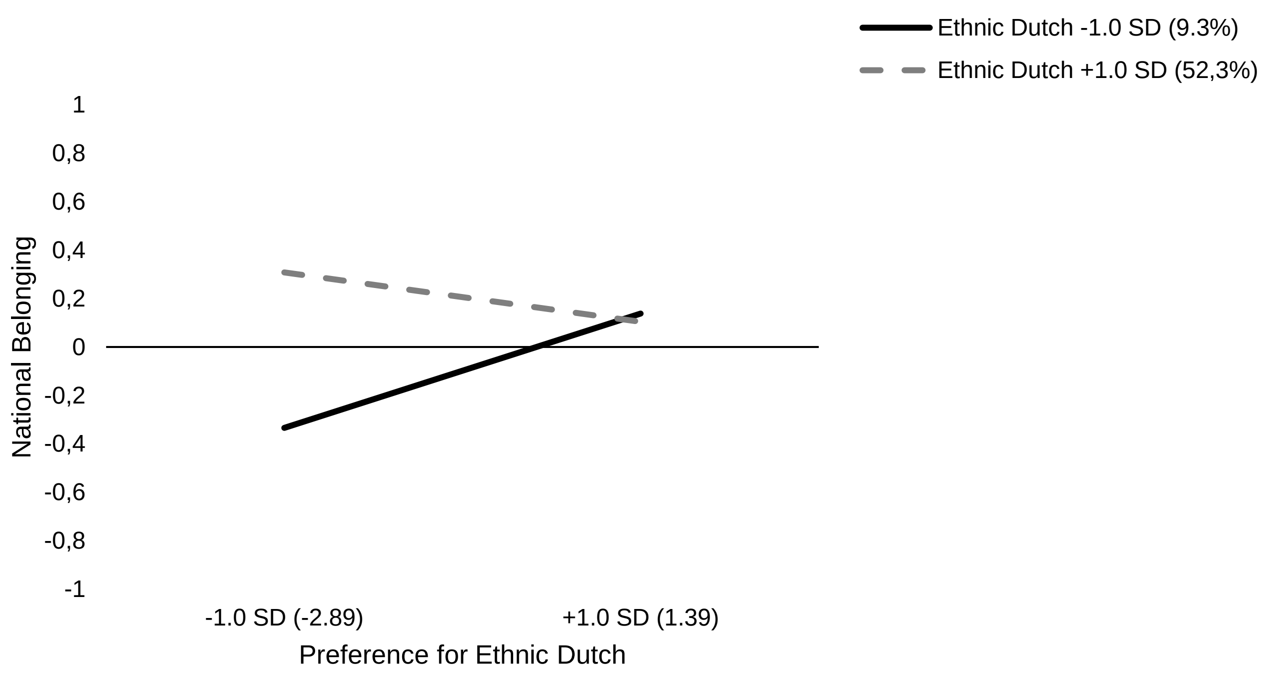

Ethnic Dutch Students

The robustness of the findings for ethnic majority group students was also checked by taking the nesting in classes into account. A similar multiple linear regression, with all parameters included, was estimated to explore which factors were predictive of ethnic Dutch students' national belonging. The results, shown in Table 2, indicated that ethnic Dutch students experienced more national belonging when they had a closer relationship with their teacher (b^* = .24, p < .001) and when they perceived their classmates as having more positive in-group norms (b^* = .30, p = .008). The other predictors and interactions included in the model were not significantly associated with ethnic Dutch students' national belonging.

Table 2

Unstandardized and Standardized Estimates for the Model Predicting Ethnic Dutch Students' National Belonging (N=212)

	Model 1			Model 2		
	B	*SE*	*b**	*B*	*SE*	*b**
% Ethnic Dutch peers	-0.12	0.20	-0.04	0.07	0.20	0.02
Teacher closeness	0.17**	0.05	0.24***	0.21***	0.05	0.29***
Perceived in-group norms	0.15**	0.05	0.30**	-	-	-
Perceived out-group norms	0.02	0.04	0.05	-	-	-
Preference towards ethnic Dutch people	-	-	-	0.03	0.03	0.07
Perceived multicultural teacher norms	0.04	0.05	0.06	0.06	0.06	0.08
% Ethnic Dutch peers x Teacher closeness	0.33	0.40	0.09	0.25	0.38	0.07
% Ethnic Dutch peers x Perceived in-group norms	0.28	0.21	0.14	-	-	-
% Ethnic Dutch peers x Perceived out-group norms	-0.09	0.14	-0.04	-	-	-
% Ethnic Dutch peers x Perceived Preference towards ethnic Dutch	-	-	-	0.00	0.13	0.00
Teacher FTE	-0.04	0.03	-0.08	-0.03	0.03	-0.06
Teacher FTE x Teacher closeness	0.07	0.04	0.11	0.06	0.04	0.08
Teacher FTE x Perceived multicultural teacher norms	0.03	0.05	0.04	0.04	0.04	0.06
Explained variance (R^2)			0.14***			0.10**

Note. * p < .05 level (2-tailed). ** p < .01 level (2-tailed). *** p < .001 level (2-tailed).

Table 2 also shows the class-nested model including the perceived preference for ethnic Dutch people on ethnic majority students' national belonging (Model 2). The results indicated that the main effect of

preference nor its interaction with classroom composition were significant predictors of ethnic Dutch students' national belonging.

Differences

To examine whether the nesting in classes affect the conclusions about the differences between the models for the ethnic minority and ethnic majority students, z-scores were calculated using the same approach as in the main analysis. The results for Model 1 indicated that the effects of the percentage of ethnic Dutch students in the classroom ($Z = 2.31$; $p = .021$) and of the interaction of percentage ethnic Dutch with teacher closeness ($Z = -2.27$; $p = .023$), differed between the two groups. The other effects did not significantly differ between groups (p-values >.05).

For Model 2, the effects of the percentage of ethnic Dutch students in the classroom ($Z = 2.07$; $p = .038$) and the interaction of percentage ethnic Dutch and teacher closeness ($Z = -2.21$; $p = .027$) also differed between the two groups. The z-score of the interaction of percentage ethnic Dutch and perceived preference for ethnic Dutch people, which was a significant predictor for ethnic minority students but not for ethnic majority students, was not significant ($Z = -1,83$; $p = .067$), as compared to the main analysis. However, the direction of this z-score is similar as to the main analysis. The other effects did not significantly differ between groups (p-values > .05).

Part Two

Results Regarding Turkish and Moroccan Students Separately

Due to sample size restrictions, the three groups of minority students were combined into one overarching category in the main analyses. To examine the possible consequences of this, the differences between Turkish, Moroccan, and Surinamese students were explored.

First, MANOVA was used to examine whether the minority groups differed from each other regarding the study variables. Results showed that there was indeed a significant overall difference, $F(10, 294)=2.15$, p = .021; Wilk's $\Lambda = 0.868$, partial $\eta^2 = .07$. Further inspection of the MANOVA results however showed that students' ethnicity only had a significant effect on national belonging ($F(2, 151) = 4.18$, p = .017; partial η^2 = .05). Turkish students reported less national belonging than Moroccan students (p = .013), but there was no significant difference in national belonging between Turkish and Surinamese students (p = .930), or between Moroccan and Surinamese students (p = .463). Note, however, that the group of Surinamese students was extremely small (N = 15).

Next, it was checked if and how, the results differed for the Turkish (N = 95) and Moroccan (N = 73) students. Given sample size restrictions, the sign of the coefficients rather than their significance was focused on.

Turkish Students

The results for Turkish students' national belonging (Table 3, Model 1) were rather similar to those in the main analyses. For example, both in the main analysis as well as for the Turkish students, the effect of the percentage of ethnic Dutch students in the classroom was positive, while the effect in both analyses was negative for the interaction between the percentage of ethnic Dutch students and teacher closeness. Interestingly, the effect of perceived in-group norms on Turkish students' national belonging was positive and significant, whilst in the main analysis this effect was positive but not significant.

It was also examined if the analysis of the Turkish students separately resulted in different outcomes regarding the second model, including students' perceived preference for ethnic Dutch people. The results are displayed in Table 3, Model 2 are similar in direction to those in the main analysis. Only the direct effect of preference towards ethnic Dutch people is negative while in the main analysis this effect is positive (B = 0.02),

but both effects are close to zero. It therefore could be that this difference in direction is due to the small regression coefficient.

Table 3

Unstandardized and Standardized Estimates for the Model Predicting Turkish Students' National Belonging (N=89)

	Model 1			Model 2		
	B	*SE*	*b**	*B*	*SE*	*b**
% Ethnic Dutch peers	1.04	0.58	0.20	1.17	0.61	0.23*
Teacher closeness	0.08	0.14	0.07	0.17	0.11	0.15
Perceived in-group norms	0.15*	0.06	0.19*	-	-	-
Perceived out-group norms	0.03	0.05	0.06	-	-	-
Preference towards ethnic Dutch people	-	-	-	-0.04	0.03	-0.08
Perceived multicultural teacher norms	0.18	0.14	0.17	0.16	0.13	0.15
% Ethnic Dutch peers x Teacher closeness	-1.09*	0.49	-0.17*	-0.75	0.41	-0.12
% Ethnic Dutch peers x Perceived in-group norms	0.77*	0.34	0.20	-	-	-
% Ethnic Dutch peers x Perceived out-group norms	-0.17	0.26	-0.06	-	-	-
% Ethnic Dutch peers x Perceived Preference towards ethnic Dutch	-	-	-	-0.45**	0.16	-0.21*
Teacher FTE	0.09	0.11	0.09	0.06	0.11	0.06
Teacher FTE x Teacher closeness	-0.15	0.12	-0.14	-0.14	0.11	-0.13
Teacher FTE x Perceived multicultural teacher norms	-0.08	0.15	-0.07	-0.06	0.14	-0.06
Explained variance (R^2)			0.25**			0.22*

Note. * p < .05 level (2-tailed). ** p < .01 level (2-tailed). *** p < .001 level (2-tailed).

Moroccan Students

As can be seen in Table 4, Model 1, the results for the Moroccan students were mostly similar compared to those the main analysis. Again the effect of the percentage of ethnic Dutch students in the classroom was positive, while the effect in both analyses was negative for the interaction between the percentage of ethnic Dutch students and teacher closeness. However, some effects appear to be different. The effects of perceived in-group norms was negative while it was positive but nonsignificant in the main analysis. Moreover, the interaction of teacher FTE and teacher closeness and the interaction of teacher FTE and perceived multicultural norms are of positive direction while they were negative but not significant in the main analysis. However, these effects are all very close to zero and not significant in both analyses.

Table 4

Unstandardized and Standardized Estimates for the Model Predicting Moroccan Students' National Belonging (N=67)

	Model 1			Model 2		
	B	*SE*	*b**	*B*	*SE*	*b**
% Ethnic Dutch peers	1.83*	0.73	0.36*	1.49*	0.66	0.29*
Teacher closeness	0.24*	0.11	0.28*	0.22*	0.10	0.25*
Perceived in-group norms	-0.08	0.08	-0.11	-	-	-
Perceived out-group norms	0.06	0.04	0.12	-	-	-
Preference towards ethnic Dutch people	-	-	-	0.10**	0.03	0.26**
Perceived multicultural teacher norms	0.08	0.12	0.09	0.07	0.12	0.08
% Ethnic Dutch peers x Teacher closeness	-1.21*	0.60	-0.18*	-1.18*	0.59	-0.17*
% Ethnic Dutch peers x Perceived in-group norms	0.02	0.39	0.01	-	-	-
% Ethnic Dutch peers x Perceived out-group norms	-0.82	0.51	-0.22	-	-	-
% Ethnic Dutch peers x Perceived Preference towards ethnic Dutch	-	-	-	-0.33	0.27	-0.15
Teacher FTE	0.18*	0.09	0.20	0.19	0.10	0.21*
Teacher FTE x Teacher closeness	0.03	0.14	0.03	0.07	0.13	0.07
Teacher FTE x Perceived multicultural teacher norms	0.11	0.13	0.11	0.10	0.13	0.10
Explained variance (R^2)			0.38**			0.37**

Note. * *p* < .05 level (2-tailed). ** *p* < .01 level (2-tailed). *** *p* < .001 level (2-tailed).

It was also examined if the analysis of the Moroccan students separately resulted in different outcomes regarding the second model, including students' perceived preference for ethnic Dutch people. The results are displayed in Table 4, Model 2 and most of the paths are of similar direction to those in the main analysis. Only the interaction effects of teacher FTE with teacher closeness and perceived teacher multicultural norms are positive while these are negative in the main analysis, but both effects are close to zero and not significant.

Turkish and Moroccan Students

With regards to the results of both models, the direction of the effects are also similar when comparing Turkish and Moroccan students. For both groups, the effects of percentage Ethnic Dutch peers and teacher closeness are positive, although they seem somewhat stronger for the Moroccan students. Moreover, for both groups, the effects of perceived out-group norms and perceived multicultural teacher norms, were positive. The interaction effects are also of similar direction in both groups. The directions of some effects did seem to differ. The effect of perceived in-group norms is positive for Turkish students, but negative for Moroccan students. The effect of perceived preference for ethnic Dutch people, on the other hand is negative for Turkish students, whilst positive for Moroccan students.
