# Supplementary material for: Classroom Predictors of National Belonging: The Role of Interethnic Contact and Teachers’ and Classmates’ Diversity Norms
Source: J Youth Adolesc. 2021 Apr 7;50(8):1709–25. doi: 10.1007/s10964-021-01430-2 (PMC8270874; doi:10.1007/s10964-021-01430-2)
Supplement: Supplementary file 2 — Supplementary Information 2 [file 10964_2021_1430_MOESM2_ESM.pdf]

Supplementary Information

Classroom Predictors of National Belonging: The Role of Interethnic Contact and Teachers' and Classmates' Diversity Norms

Journal of Youth and Adolescence

Contents:

Part One: Detailed Description of Measurement Invariance Analysis

Part Two: Mplus Syntax Used for the Alignment Method

Part One

Detailed Description of Measurement Invariance Analysis

Because the goal was to compare minority and ethnic majority group students, it was investigated whether the scales for national belonging, teacher closeness, and perceived multicultural teacher norms were scalar invariant by using the Alignment Method (Asparouhov & Muthén, 2014). The alignment method identifies a model with the least measurement noninvariance and with the best possible fit, amongst all possible multigroup models. The alignment method comprises of two steps. The first step is to fit a standard configural invariance model, where the factor loadings and intercepts are allowed to differ between groups and the factor means and factor variances are fixed to zero and one respectively. This model has the most optimal fit. In the second step the actual alignment analysis is performed by freeing the factor means and variances in both groups and estimating their values using a simplicity function that minimizes the total amount of noninvariance over all parameters. This simplicity function is similar to rotation criteria used with exploratory factor analysis and does not compromise the fit of the model (Asparouhov & Muthén, 2014). In running the alignment optimization, two options can be chosen: Free and Fixed. It is recommended to first run the free optimization as this works best when there is a large degree of noninvariance. If this option does not converges, the fixed alignment optimization should be chosen, which fixes the factor mean of the group with the smallest latest factor means to 0 (Asparouhov & Muthén, 2014).

The results of the alignment method are presented below. In the first step, a conventional configural model was run to determine whether the alignment method is possible to use. Model fit indices indicate a good model fit for this configural model ($\chi^2(102) = 94.39$, $p = .691$; RMSEA = .000; CFI = 1.00; TLI = 1.008; SRMR = .045) which means that the alignment method can be used.

In the second step, the alignment method was estimated using the “Fixed” option as the “Free” option did not converged. With this option the factor mean of the group with the smallest mean, in this case the minority group, was fixed to 0. In Table 1, results of the alignment analysis are shown. For each parameter (intercept and loadings) the fit function contribution shows the amount of noninvariance. The lower the contribution value, the more invariant the parameter is (Asparouhov & Muthén, 2014). In the second column of Table 1, the R^2 value indicates the amount of noninvariance that can be absorbed by the group-varying factor means and variances. A value closer to 1, indicates a higher degree of invariance (Asparouhov & Muthén, 2014). In third column the number of groups across which the parameter is invariant is shown.

Table 1

Alignment Fit Statistics for Each Parameter

	Intercepts			Loadings		
Item	*Fit Function Contribution*	R^2	*# groups with approximate measurement invariance*	*Fit Function Contribution*	R^2	*# of groups with approximate measurement invariance*
National belonging						
NB1	-0.500	.992	2	-0.482	.910	2
NB2	-0.318	1.000	2	-0.316	1.000	2
NB3	-0.386	.997	2	-0.371	.947	2
Teacher closeness						
RelTeach1	-0.359	.996	2	-0.509	.691	2
RelTeach2	-0.347	.980	2	-0.341	.800	2
RelTeach3	-0.397	.981	2	-0.518	.679	2
RelTeach4	-0.499	.000	2	-0.325	.955	2
RelTeach5	-0.359	.945	2	-0.511	.000	2
RelTeach6	-0.354	.947	2	-0.321	.987	2
Perceived multicultural teacher norms						
TDivNorm1	-0.421	.894	2	-0.317	.999	2
TDivNorm2	-0.391	.582	2	-0.319	.995	2
TDivNorm3	-0.316	1.000	2	-0.321	.984	2

As can be seen in table one, the fit function contribution values for both loadings and intercepts are relatively small, indicating more invariance for the parameters across groups. Moreover, for most of the parameters (intercepts and loadings) the R^2 is close to one, indicating a higher degree of invariance. There are also some parameters with a lower R^2 value (e.g., the intercept of TDivNorm2, or the loadings of Relteach1, and Relteach3) or an R^2 value of 0 (the intercept of Relteach4 and the loading of Relteach5). However, this is not uncommon as this value can be lower even if the other results show that the parameter is invariant across groups. This is most likely due to a small factor mean variability or a small loading (Asparouhov, 2016). Finally, Table 1 shows that for all parameters approximate measurement invariance holds across all of the groups.

Furthermore, in Table 2 results for each parameter (intercepts and loadings) with regards to the differences in values between groups and whether this difference is significant is indicated. As can be seen, the are no significant differences between the groups on any of the intercepts or factor loadings. This offers further support for the inference that approximate measurement invariance holds across groups.

Table 2

Values for Intercepts and Loadings per Parameter and Differences Between the Groups

	Intercepts					Loadings				
Item	*Group 1* *Value*	*Group 0* *Value*	*Difference*	*S.E.*	*p*	*Group 1* *Value*	*Group 0* *Value*	*Difference*	*S.E.*	*p*
National belonging										
NB1	2.776	2.953	-0.177	0.149	.234	1.054	0.843	0.211	0.143	.139
NB2	2.449	2.461	-0.012	0.018	.494	1.038	1.036	0.002	0.022	.932
NB3	2.818	2.735	0.082	0.134	.541	0.903	0.955	-0.092	0.127	.468
Teacher closeness										
RelTeach1	3.008	3.069	-0.061	0.099	.538	0.908	0.711	0.196	0.137	.151
RelTeach2	1.726	1.678	0.048	0.086	.573	0.769	0.817	-0.047	0.112	.672
RelTeach3	2.831	2.919	-0.088	0.094	.348	0.882	0.684	0.198	0.120	.098
RelTeach4	2.700	2.502	0.198	0.135	.142	0.936	0.968	-0.032	0.128	.801
RelTeach5	2.885	2.940	-0.055	0.099	.579	0.655	0.834	-0.179	0.144	.215
RelTeach6	2.296	2.245	0.051	0.084	.545	0.751	0.732	0.019	0.077	.807
Perceived multicultural teacher norms										
TDivNorm1	2.507	2.369	0.139	0.145	.341	1.059	1.051	0.008	0.134	.955
TDivNorm2	2.746	2.851	-0.105	0.131	.423	1.023	1.004	0.018	0.125	.883
TDivNorm3	2.438	2.439	-0.001	0.011	.930	0.898	0.921	-0.024	0.127	.852

Finally, the alignment method computes the average invariance index. This is the average R^2 across all parameters and indicates the degree of confidence to which means can be meaningfully compared across the groups in the data. In general, a value of 1 represents perfect scalar invariance, whereas 0 represents full non-invariance. In this case, the average invariance index is R^2=.84. This indicates that there is a high degree of confidence with which the means of variables can be compared across the minority and ethnic majority groups in the current study.

One of the drawbacks of the alignment method is that the results are tied to the specific dataset and the method can find measurement invariance when there is not when using small sample sizes. Given the relatively small sample size, a Monte Carlo (MC) simulation study was included to check the external validity and the quality of the alignment solution as well (Asparouhov & Muthén, 2014). Given the sample sizes (Ethnic majority group *N*=213; Minority group *N*=183) simulations with an N of 150, 200, and 250 with 500 replications were ran. Simulations were based on the starting values retrieved from the alignment results. Syntax for the alignment method and the simulations can be found in Part Two of this supplementary information. In Table 3, the correlations and mean square error of the population and estimate values are shown. These represent the correlations between the estimated and actual factor means and variances per replication, which are then averaged over the 500 replications. These correlations represent not only how well the alignment method works given the type of non-invariance in the data but also given the sample size. According to Muthén and Asparouhov (2018) these correlations should preferably not be below .98 and never below .95 for the alignment method to be reliable. As can be seen in Table 3, these correlations for the factor means are all above the cut-off in all simulations. However, regarding the factor variances for the simulations with a lower sample size, some of these correlations are below the .95 cut-off, although still not very low (>.90). Based on these results, the alignment results are trustworthy, even with somewhat smaller sample sizes. The mean square error reported in Table 3, further indicates how well the actual and estimated values match. Ideally these values should be close to 0, however no clear cut-off is given. As can be seen in Table 3, the mean square errors for the mean and variances of all scales are close to zero for all simulations.

Table 3

Correlations and Mean Square Error of Population and Estimate Values for all Simulations

Monte Carlo design	*Correlations*						*Mean Square Error*					
	n=150		*n=200*		*n=250*		*n=150*		*n=200*		*n=250*	
	Ave-rage	*SD*	Ave-rage	*SD*	Ave-rage	*SD*	Ave-rage	*SD*	Ave-rage	*SD*	Ave-rage	*SD*
National Belonging												
Mean	1.00	0.00	1.00	0.00	1.00	0.00	0.07	0.06	0.06	0.05	0.06	0.04
Variance	1.00	0.00	1.00	0.00	1.00	0.00	0.04	0.03	0.04	0.03	0.04	0.03
Teacher closeness												
Mean	1.00	0.00	1.00	0.00	1.00	0.00	0.08	0,06	0.07	0.06	0.06	0.05
Variance	0.92	0.38	0.96	0.28	0.96	0.28	0.10	0.08	0.09	0.07	0.08	0.06
Perceived multicultural teacher norms												
Mean	0.98	0.18	0.98	0.18	1.00	0.00	0.08	0.06	0.07	0.05	0.06	0.04
Variance	0.94	0.34	0.97	0.24	0.99	0.15	0.9	0.07	0.08	0.06	0.07	0.05

References

Asparouhov, T. (2016). *Multigroup alignment method*. http://www.statmodel.com/discussion/messages/9/13900.html?1497121840

Asparouhov, T., & Muthén, B. O. (2014). Multiple-group factor analysis alignment. *Structural Equation Modeling, 21*(4), 495–508. https://doi.org/10.1080/10705511.2014.919210

Muthén, B. O., & Asparouhov, T. (2018). Recent Methods for the Study of Measurement Invariance With Many Groups: Alignment and Random Effects. *Sociological Methods and Research, 47*(4), 637–664. https://doi.org/10.1177/0049124117701488

Part Two

Mplus Syntax Used for the Alignment Method

<u>Step 1: Configural factor model.</u>

```
Title: Measurement invariance_configural factorial invariance_Full;

data: file =; !Insert datafile name

variable: names = schoolnr classnr teachnr studnr parSES1 parSES2 parSES3
        parSES4 relTeac1 relTeac3 relTeac5 relTeac7 relTeac9 relTea10
        clAttSur clAttNL clAttTrk clAttMor multTch1 multTch2 multTch3
        NB1 NB2 NB3 MinGr;
      USEVAR = relTeac1 relTeac3 relTeac5 relTeac7 relTeac9 relTea10
      multTch1 multTch2 multTch3 NB1 NB2 NB3 MinGr;
      Grouping = MinGr(0=Ethnic_Dutch 1=Minority_students);
      missing = all(-999);

Analysis: ESTIMATOR = MLR;

MODEL: NB BY NB1* NB2 NB3;
     NB@1;
    [NB@0];
    Close BY relTeac1* relTeac3 relTeac5 relTeac7 relTeac9 relTea10;
    Close@1;
    [Close@0];
    TDivNorm BY multTch1* multTch2 multTch3;
    TDivNorm@1;
    [TDivNorm@0];

MODEL Ethnic_Dutch:
    NB BY NB1* NB2 NB3;
    NB@1;
    [NB@0];
    Close BY relTeac1* relTeac3 relTeac5 relTeac7 relTeac9 relTea10;
    Close@1;
    [Close@0];
    TDivNorm BY multTch1* multTch2 multTch3;
    TDivNorm@1;
    [TDivNorm@0];
    [relTeac1-NB3];

Output: TECH4 MODINDICES(ALL 0) sampstat STDYX;
```

<u>Step 2a: Alignment Method, Free optimization</u>

```
Title: Measurement invariance_Alignment_Full_Free;

data: file =; !Insert datafile name

variable: names = schoolnr classnr teachnr studnr parSES1 parSES2 parSES3
        parSES4 relTeac1 relTeac3 relTeac5 relTeac7 relTeac9 relTea10
```

```
      clAttSur clAttNL clAttTrk clAttMor multTch1 multTch2 multTch3
      NB1 NB2 NB3 MinGr;
     USEVAR = relTeac1 relTeac3 relTeac5 relTeac7 relTeac9 relTea10
     multTch1 multTch2 multTch3 NB1 NB2 NB3 MinGr;
     classes = c(2);
     KNOWNCLASS = c(MinGr=0 1);
     missing = all(-999);

Analysis: TYPE = MIXTURE;
     ESTIMATOR = MLR;
     ALIGNMENT = Free;

MODEL:
  %OVERALL% !it means the CFA model specified below is applicable in every group
   NB BY NB1* NB2 NB3;
   Close BY relTeac1* relTeac3 relTeac5 relTeac7 relTeac9 relTea10;
   TDivNorm BY multTch1* multTch2 multTch3;

Output: tech8 align SVALUES;

SAVEDATA:
FILE IS alignment_factor_scores.dat;
FORMAT IS FREE;
SAVE = FSCORES;
```

Step 2b: Alignment Method, Fixed optimization (used in appendix)

```
Title: Measurement invariance_Alignment_Full_Free;

data: file = ; !Insert datafile name

variable: names = schoolnr classnr teachnr studnr parSES1 parSES2 parSES3
      parSES4 relTeac1 relTeac3 relTeac5 relTeac7 relTeac9 relTea10
      clAttSur clAttNL clAttTrk clAttMor multTch1 multTch2 multTch3
      NB1 NB2 NB3 MinGr;
     USEVAR = relTeac1 relTeac3 relTeac5 relTeac7 relTeac9 relTea10
     multTch1 multTch2 multTch3 NB1 NB2 NB3 MinGr;
     classes = c(2);
     KNOWNCLASS = c(MinGr=0 1);
     missing = all(-999);

Analysis: TYPE = MIXTURE;
     ESTIMATOR = MLR;
     ALIGNMENT = Fixed(1);

MODEL:
  %OVERALL% !it means the CFA model specified below is applicable in every group
   NB BY NB1* NB2 NB3;
   Close BY relTeac1* relTeac3 relTeac5 relTeac7 relTeac9 relTea10;
   TDivNorm BY multTch1* multTch2 multTch3;

Output: tech8 align SVALUES;

SAVEDATA:
```

```
FILE IS alignment_factor_scores.dat;
FORMAT IS FREE;
SAVE = FSCORES;
```

Step 3: Simulation studies

```
Title: Measurement invariance_NB only_Simulation_Alignment;

MONTECARLO:
NAMES = NB1 NB2 NB3 relteac1 relteac3 relteac5 relteac7
    relteac9 reltea10 multtch1 multtch2 multtch3; ! Names of indicator variables (only)
ngroups = 2; ! Your number of groups
NOBSERVATIONS = 2(150); ! This is again a number of groups and sample size of
    !each group in parentheses; change to 200 and 250 for the other simulation results.
NREPS = 500; ! This is how many times the data generation and analysis should be repeated.
SEED = 01092020;

ANALYSIS:
TYPE = MIXTURE;
ESTIMATOR = MLR;
alignment = fixed(2);

MODEL POPULATION:

  %OVERALL%

  nb BY nb1*1;
  nb BY nb2*1;
  nb BY nb3*1;
  close BY relteac1*1;
  close BY relteac3*1;
  close BY relteac5*1;
  close BY relteac7*1;
  close BY relteac9*1;
  close BY reltea10*1;
  tdivnorm BY multtch1*1;
  tdivnorm BY multtch2*1;
  tdivnorm BY multtch3*1;

  %G#1%

  nb BY nb1*0.84260;
  nb BY nb2*1.03598;
  nb BY nb3*0.99508;
  close BY relteac1*0.71138;
  close BY relteac3*0.81674;
  close BY relteac5*0.68412;
  close BY relteac7*0.96808;
  close BY relteac9*0.83420;
  close BY reltea10*0.73181;
  tdivnorm BY multtch1*1.05131;
  tdivnorm BY multtch2*1.00430;
  tdivnorm BY multtch3*0.92136;
```

```
close WITH nb*0.11467;
tdivnorm WITH nb*0.04102;
tdivnorm WITH close*0.09583;

[ relteac1*3.06862 ];
[ relteac3*1.67787 ];
[ relteac5*2.91899 ];
[ relteac7*2.50238 ];
[ relteac9*2.94001 ];
[ reltea10*2.24518 ];
[ multtch1*2.36895 ];
[ multtch2*2.85120 ];
[ multtch3*2.43900 ];
[ nb1*2.95339 ];
[ nb2*2.46091 ];
[ nb3*2.73547 ];
[ nb*0.88378 ];
[ close*0.39477 ];
[ tdivnorm*-0.27056 ];

relteac1*0.44071;
relteac3*0.95835;
relteac5*0.40180;
relteac7*0.66234;
relteac9*0.52666;
reltea10*0.74713;
multtch1*0.52442;
multtch2*0.75143;
multtch3*0.74069;
nb1*0.09994;
nb2*0.32079;
nb3*0.08126;
nb*0.34571;
close*0.67785;
tdivnorm*0.63197;

%G#2%

nb BY nb1*1.05394;
nb BY nb2*1.03790;
nb BY nb3*0.90301;
close BY relteac1*0.90788;
close BY relteac3*0.76928;
close BY relteac5*0.88238;
close BY relteac7*0.93577;
close BY relteac9*0.65547;
close BY reltea10*0.75064;
tdivnorm BY multtch1*1.05890;
tdivnorm BY multtch2*1.02267;
tdivnorm BY multtch3*0.89773;

close WITH nb*0.30784;
tdivnorm WITH nb*0.22708;
```

```
    tdivnorm WITH close*0.40909;

    [ relteac1*3.00758 ];
    [ relteac3*1.72624 ];
    [ relteac5*2.83082 ];
    [ relteac7*2.69997 ];
    [ relteac9*2.88515 ];
    [ reltea10*2.29616 ];
    [ multtch1*2.50748 ];
    [ multtch2*2.74600 ];
    [ multtch3*2.43802 ];
    [ nb1*2.77627 ];
    [ nb2*2.44886 ];
    [ nb3*2.81759 ];
    [ nb*0 ];
    [ close*0 ];
    [ tdivnorm*0 ];

    relteac1*0.62233;
    relteac3*1.46537;
    relteac5*0.56671;
    relteac7*1.01245;
    relteac9*1.02081;
    reltea10*1.05519;
    multtch1*0.48842;
    multtch2*0.65267;
    multtch3*1.13411;
    nb1*0.20969;
    nb2*0.41049;
    nb3*0.32505;
    nb*1;
    close*1;
    tdivnorm*1;



MODEL:
      %OVERALL%

    nb BY nb1*1;
    nb BY nb2*1;
    nb BY nb3*1;
    close BY relteac1*1;
    close BY relteac3*1;
    close BY relteac5*1;
    close BY relteac7*1;
    close BY relteac9*1;
    close BY reltea10*1;
    tdivnorm BY multtch1*1;
    tdivnorm BY multtch2*1;
    tdivnorm BY multtch3*1;

    %G#1%
```

```
nb BY nb1*0.84260;
nb BY nb2*1.03598;
nb BY nb3*0.99508;
close BY relteac1*0.71138;
close BY relteac3*0.81674;
close BY relteac5*0.68412;
close BY relteac7*0.96808;
close BY relteac9*0.83420;
close BY reltea10*0.73181;
tdivnorm BY multtch1*1.05131;
tdivnorm BY multtch2*1.00430;
tdivnorm BY multtch3*0.92136;

close WITH nb*0.11467;
tdivnorm WITH nb*0.04102;
tdivnorm WITH close*0.09583;

[ relteac1*3.06862 ];
[ relteac3*1.67787 ];
[ relteac5*2.91899 ];
[ relteac7*2.50238 ];
[ relteac9*2.94001 ];
[ reltea10*2.24518 ];
[ multtch1*2.36895 ];
[ multtch2*2.85120 ];
[ multtch3*2.43900 ];
[ nb1*2.95339 ];
[ nb2*2.46091 ];
[ nb3*2.73547 ];
[ nb*0.88378 ];
[ close*0.39477 ];
[ tdivnorm*-0.27056 ];

relteac1*0.44071;
relteac3*0.95835;
relteac5*0.40180;
relteac7*0.66234;
relteac9*0.52666;
reltea10*0.74713;
multtch1*0.52442;
multtch2*0.75143;
multtch3*0.74069;
nb1*0.09994;
nb2*0.32079;
nb3*0.08126;
nb*0.34571;
close*0.67785;
tdivnorm*0.63197;

%G#2%

nb BY nb1*1.05394;
```

```
nb BY nb2*1.03790;
nb BY nb3*0.90301;
close BY relteac1*0.90788;
close BY relteac3*0.76928;
close BY relteac5*0.88238;
close BY relteac7*0.93577;
close BY relteac9*0.65547;
close BY reltea10*0.75064;
tdivnorm BY multtch1*1.05890;
tdivnorm BY multtch2*1.02267;
tdivnorm BY multtch3*0.89773;

close WITH nb*0.30784;
tdivnorm WITH nb*0.22708;
tdivnorm WITH close*0.40909;

[ relteac1*3.00758 ];
[ relteac3*1.72624 ];
[ relteac5*2.83082 ];
[ relteac7*2.69997 ];
[ relteac9*2.88515 ];
[ reltea10*2.29616 ];
[ multtch1*2.50748 ];
[ multtch2*2.74600 ];
[ multtch3*2.43802 ];
[ nb1*2.77627 ];
[ nb2*2.44886 ];
[ nb3*2.81759 ];
[ nb*0 ];
[ close*0 ];
[ tdivnorm*0 ];

relteac1*0.62233;
relteac3*1.46537;
relteac5*0.56671;
relteac7*1.01245;
relteac9*1.02081;
reltea10*1.05519;
multtch1*0.48842;
multtch2*0.65267;
multtch3*1.13411;
nb1*0.20969;
nb2*0.41049;
nb3*0.32505;
nb*1;
close*1;
tdivnorm*1;
```
